# Maintenance of Sensitivity of the T-SPOT.*TB* Assay after Overnight Storage of Blood Samples, Dar es Salaam, Tanzania

**DOI:** 10.1155/2012/345290

**Published:** 2012-02-06

**Authors:** Elizabeth A. Talbot, Isaac Maro, Katherine Ferguson, Lisa V. Adams, Lillian Mtei, Mecky Matee, C. Fordham von Reyn

**Affiliations:** ^1^Dartmouth Medical School, Hanover, NH 03755, USA; ^2^Infectious Diseases and International Health Section, 1 Medical Center Drive, Lebanon, NH 03756, USA; ^3^Muhimbili University of Health and Allied Sciences, Dar es Salaam, Tanzania; ^4^Dartmouth College, Hanover, NH 03755, USA

## Abstract

*Background*. T-SPOT.*TB* is an interferon gamma release assay for detecting *Mycobacterium tuberculosis* infection. The requirement to process within 8 hours is constraining, deters use, and leads to invalid results. Addition of T Cell *Xtend* reagent may allow delayed processing, but has not been extensively field tested. *Design*. Consecutive AFB smear positive adult tuberculosis patients were prospectively recruited in Dar es Salaam, Tanzania. Patients provided a medical history, 1–3 sputum samples for culture and 1 blood sample which was transported to the laboratory under temperature-controlled conditions. After overnight storage, 25 **μ**L of T Cell *Xtend* reagent was added per mL of blood, and the sample was tested using T-SPOT.*TB*. *Results*. 143 patients were enrolled: 57 patients were excluded because temperature control was not maintained, 19 patients were excluded due to red blood cell contamination, and one did not provide a sputum sample for culture. Among 66 evaluable patients, overall agreement between T-SPOT.*TB* and culture was 95.4% (95%CI; 87.1–99.0%) with Kappa value 0.548. Sensitivity of T-SPOT.*TB* when using T Cell *Xtend* reagent was 96.8% (95%CI; 88.8–99.6%). *Conclusions*. When T Cell *Xtend* reagent is added to specimens held overnight at recommended temperatures, T-SPOT.*TB* is as sensitive as the standard assay in patients with tuberculosis.

## 1. Introduction

The T-SPOT.*TB* (Oxford Immunotec Ltd, Abingdon, UK) is an interferon gamma release assay (IGRA) for detection of latent *Mycobacterium tuberculosis* infection (LTBI). This assay detects T-lymphocytes specific for *M. tuberculosis* antigens that are absent from *M. bovis* BCG and most environmental mycobacteria [1]. Currently, T-SPOT.*TB* must be performed within 8 hours of sample collection. Logistically, this requires that patients have blood drawn early enough to transport the sample to the laboratory, which must assay samples on the day of receipt. This precludes batching samples, which would save resources and testing supplies. Moreover, in many countries, laboratories are centralized and are not in close proximity to where the blood sample is drawn. Therefore, it is not possible to transport samples to arrive at the receiving laboratory for processing on the same day. 

To allow greater test processing flexibility, the manufacturer of T-SPOT.*TB* developed a proprietary reagent called T Cell *Xtend*, which they report can be added to whole blood samples stored at 10–25°C to increase this timeframe up to 32 hours [2].

## 2. Materials and Methods

The ethical review boards of Dartmouth Medical School (Hanover NH), the National Institute for Medical Research (Dar es Salaam, Tanzania) and Muhimbili University of Health and Allied Sciences (MUHAS, Dar es Salaam, Tanzania) approved the study. The study was conducted according to the principles of good clinical practice and under the International Conference on Harmonization guidelines.

### 2.1. Participant Enrolment

All AFB smear positive individuals referred for initiation of TB treatment to one of two National Tuberculosis and Leprosy Programme (NTLP) TB treatment clinics in Dar es Salaam, Tanzania, were approached by a trained study nurse to determine their interest in being included in the study. The study nurse explained the study and answered any questions in the patient's native language. The participants then signed the approved informed consent or, in the presence of a witness, indicated their signature with a fingerprint. All participants provided a medical history, up to three sputum samples and a blood sample for testing by IGRA and were offered HIV testing according to the NTLP guidelines.

### 2.2. IGRA Testing

A single tube of blood was drawn from each participant. During the initial enrolment period, blood samples were transported to the laboratory under ambient temperature conditions (maximum average temperatures during the enrolment months were 28°C for September and 29°C for October) and, in the latter enrolment period, under temperatures ensured as <25°C by use of a temperature control shipping box (GreenBox Thermal Management System (Thermosafe Brands, Arlington Heights, IL, USA)). The blood samples were then stored overnight in an air conditioned laboratory (<25°C) and tested using T-SPOT.*TB* according to the manufacturer's instructions for use [3]. T Cell *Xtend* reagent was added to each blood sample at 25 *μ*L/mL blood, mixed and incubated for 20 minutes at room temperature before processing. Peripheral blood mononuclear cells (PBMC) were harvested by Ficoll density gradient centrifugation using Leucosep tubes (Oxford Immunotec, Abingdon, UK). The PBMCs were washed, counted using a hemocytometer, and plated at 2.5 × 10^5^ cells per well into a membrane-bottomed plate, coated with anti-interferon gamma antibody. PBMCs were incubated overnight in the presence of the provided TB antigens (ESAT-6 and CFP10) along with controls (positive mitogen control and a nil control). The PBMCs producing interferon gamma were revealed as spots by incubation with an enzyme conjugated secondary antibody for interferon gamma and a colour producing enzyme substrate. Spots were counted, and clinical result was recorded according to the approved algorithm where, compared to the nil control, 6 spots and above are positive and 5 spots and below are negative. Results with a low-mitogen response (<20 spots) or a high-nil control response (>10 spots) were recorded as invalid.

### 2.3. Mycobacterial Studies

AFB smears were done according to the Ziehl-Neelsen method by trained research personnel. Sputum was cultured for mycobacteria on Lowenstein Jensen slants.

### 2.4. Statistical Analyses

Sensitivity was calculated as the number of T-SPOT.*TB* positive samples divided by the number of culture positive samples multiplied by 100. 95% confidence limits were calculated. The Kappa statistic was used to measure overall agreement between culture and T-SPOT.*TB* results. Analyses were conducted using Excel.

## 3. Results

**Figure 1:**
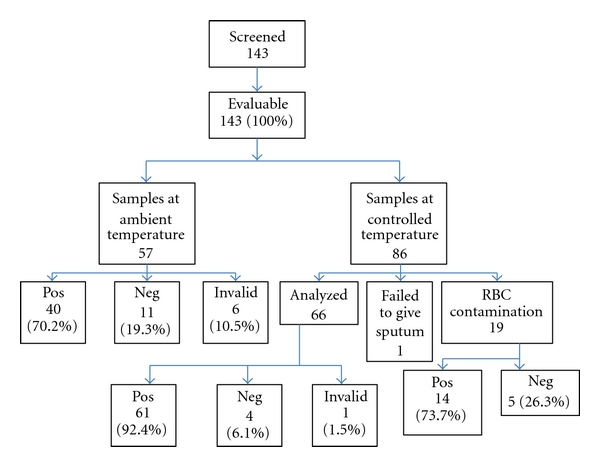
Patient flow and testing results using T-SPOT.*TB* and T Cell *Xtend, *Dar es Salaam, Tanzania.

A total of 143 participants were enrolled into the study (Figure 1). The initial 57 blood samples drawn at one clinic were transported and stored at ambient temperatures prior to processing. After processing, it was recognized that these specimens were not reliably kept at temperatures below 25°C (the required temperature per protocol), and therefore these 57 samples were excluded from the final sensitivity analysis. Temperature control measures were instituted for the subsequent collection of samples from an additional 86 participants. Nineteen of these 86 (22.1%) participants were excluded because the laboratorian observed red blood cell contamination in the PBMC layer so the PBMCs could not be accurately counted. One of 86 (1.2%) participants failed to provide a sputum sample for culture and was excluded.

Therefore, 66 results were available for analysis. The participants were all black African and ranged in age from 18–60 years. The majority were male and BCG vaccinated (71% and 77%, resp.); 17 (26%) were HIV-positive, 24 (36%) were HIV-negative, and 25 (38%) declined testing.

 Of the 66 T-SPOT.*TB* results, 61 (92.4%) were positive, 4 (6.1%) were negative, and 1 (1.5%) was invalid. The overall agreement between the 65 valid T-SPOT.*TB* and culture results was 95.4% (62/65: 95%CI; 87.1–99.0%) with a Kappa value of 0.548. The sensitivity of the T-SPOT.*TB* assay when run after delayed processing using the T Cell Xtend reagent was 96.8% (60/62: 95%CI; 88.8–99.6%). Among 41 T-SPOT.*TB* results from participants with known HIV status, T-SPOT.*TB* results were positive in 14 (82.4%) of those who were HIV-positive and 23 (95.8%) of those who were HIV-negative (*P* > 0.05). However, limiting to valid results from culture positive patients, the sensitivity of the assay among samples from HIV-positive participants was 92.9% (13/14).

In spite of exclusion because of protocol noncompliance, results from the 57 samples transported at ambient temperature were also analyzed. Of the 57, 40 (70.2%) were positive, 11 (19.3%) were negative, and 6 (10.5%) was invalid. Compared with the results from the 66 samples with temperature control, false negative results among these 57 samples were more common (*P* = 0.05). Results from 19 samples were excluded because of red blood cell contamination showed 14 (73.7%) positive and 5 (26.3%) negative. Compared with the results from 66 samples with temperature control, false negative results among these 19 were statistically significantly more common (*P* = 0.04). 

## 4. Discussion

T-SPOT.*TB* with delayed processing using T Cell *Xtend* among patients with culture-confirmed tuberculosis showed high sensitivity, comparable to that reported for immediate processing. In a recent meta-analysis of 14 studies of T-SPOT.*TB*, (without *Xtend*) in low- and middle-income countries, Metcalfe et al. reported sensitivity of 83% (95% CI, 63–94%) among HIV-positive and -negative tuberculosis patients [4].

This high sensitivity we observed is also consistent with pilot studies with the T Cell *Xtend* reagent completed at three clinical sites including the UK and South Africa [5]. Lenders et al. studied the utility of T Cell *Xtend,* conducting T-SPOT.*TB* assays the same day as collection and approximately one and two days after collection [6]. Spot counts from 66 specimens T-SPOT.*TB* assayed without the T Cell *Xtend* reagent one day after collection were higher than those processed on the day of collection. In contrast, spot counts from 215 specimens assayed two days after collection with the T Cell *Xtend* reagent were similar to those processed on the day of collection. The T Cell *Xtend* reagent reduced the proportion of samples that changed from positive to negative and vice versa (4.54% to 2.83%), but this was not statistically significant [6]. Wang et al. had similar high agreement (98.2%) among 108 specimens of results of immediate T-SPOT.*TB* and T-SPOT.*TB* with the T Cell *Xtend* reagent processed 23–32 hours after collection [7].

In one of our patients, T-SPOT.*TB* was positive and culture was negative (Table 1). This patient was an HIV-infected male, who submitted three sputum specimens which were 3+, 4+ and 1+ AFB smear positive, but all cultures were negative. The T-SPOT.*TB *result was interpreted as positive, given that the Nil control well showed 6 spots, Panel A 6, and Panel B 15. Per the package insert, the test result is positive if Panel A minus Nil control and/or Panel B minus Nil control is 6 spots [3]. It may be that the T-SPOT.*TB *result was falsely positive, or that the culture was falsely negative, perhaps because the patient had received antituberculosis therapy. We do not have follow-up data on the patient to know if they demonstrated a typical response to antituberculosis therapy, or there was an alternative diagnosis made.

Our samples transported using a validated system for temperature control at or below 25°C showed sensitivity that is equivalent to that reported for the same day T-SPOT.*TB* assay [1, 3]. However, our study clearly illustrates the need to control temperature. In sub-Sahara African settings, a simple solution for shipment over long distances would be required, such as the use of an insulated package system.

Red blood cell contamination also interferes with assay reliability because the presence of large numbers of red blood cells makes accurate counting of the PBMC fraction difficult, and therefore the required number of PBMCs cannot be reliably determined. Red blood cell contamination is more common when a hemocytometer is used (as in this study), and the use of automated instruments that provide a differential count of white blood cells would eliminate this problem.

## 5. Conclusions

T-SPOT.*TB* can be run on blood samples not contaminated with red blood cells and maintained overnight by the use of T Cell *Xtend* reagent without affecting the sensitivity of the assay. Storage and transport of samples at temperatures <25°C are critical to obtaining optimum sensitivity, and use of an automated cell counting instrument may correct the reduced sensitivity observed with red blood cell contamination.

## Figures and Tables

**Table 1:** Overall agreement between TB culture and T-SPOT.*TB*.

		T-SPOT.*TB *		
		Positive	Negative	Invalid
Culture	Positive	60	2	1*
	Negative	1	2	0

*1/66 (1.5%) of T-SPOT.TB results was invalid due to a high nil control result.
